# Prevalence and correlates of suicidal behaviour among a national population-based sample of adults in Zambia

**DOI:** 10.4102/sajpsychiatry.v27i0.1566

**Published:** 2021-01-13

**Authors:** Supa Pengpid, Karl Peltzer

**Affiliations:** 1 ASEAN Institute for Health Development, Mahidol University, Salaya, Thailand; 2 Department of Research Administration and Development, University of Limpopo, Polokwane, South Africa

**Keywords:** adults, correlates, suicide attempt, suicidal behaviour, Zambia

## Abstract

**Background:**

Suicide is a major public health issue.

**Aim:**

This study aimed to assess the prevalence and correlates of lifetime suicide attempts and past 12-month suicidal behaviour (ideation, plans and/or attempts) among adults (18–69 years).

**Setting:**

Zambia.

**Method:**

National cross-sectional data from 4302 adults (median age: 31 years) who took part in the ‘2017 Zambia STEPS survey’ were analysed.

**Results:**

The results indicate that 2.3% of participants had ever attempted suicide and 8.5% engaged in past 12-month suicidal behaviour (ideation 7.8%, plan 3.6% and/or attempt 1.1%). In adjusted logistic regression analysis, having a family member who died from suicide, having had a heart attack, angina or stroke and daily tobacco smoking were associated with ever suicide attempt. In adjusted logistic regression, female sex, non-paid work status (including student, homeworker and retired), alcohol use-related family problem, passive smoking, heart attack, angina or stroke and alcohol dependence were positively associated with past 12-month suicidal behaviour, and belonging to other ethnic groups was negatively associated with past 12-month suicidal behaviour. In addition, in an unadjusted analysis, 18–24-year-old participants, those who were never married, separated, divorced or widowed, having urban residence, family members died from suicide, having lower systolic blood pressure and daily smokeless tobacco use, were associated with past 12-month suicidal behaviour.

**Conclusion:**

Almost 1 in 10 participants was engaged in suicidal behaviour in the past 12 months, and several associated indicators were found that can assist in planning interventions.

## Introduction

Every year almost 800 000 persons die from suicide, and four in five of them are from low- and middle-income countries, making it an important public health concern.^1^ For every death by suicide, there are numerous more individuals making suicide attempts.^1^ ‘Each suicide attempt carries the possibility of subsequent attempts of increasing lethality, significant psychological suffering, long-term physical injury and death, as well as lasting negative effects on families and communities’.^2,3^ Suicide prevention efforts need to build on the epidemiological profile of suicidal behaviour.^1^

People with mental disorders, such as depression and alcohol use disorders,^1^ and with chronic illness, such as epilepsy, cardiovascular disease and diabetes,^4,5,6,7,8^ hypercholesterolemia^7,9^ and low blood pressure,^10^ have been found to be at a higher risk for suicidal behaviour. Experiencing adverse life events,^1,11,12^ unemployment and lower socio-economic status,^4,5,6^ female sex, younger age and a family history of suicide^2,3,13^ have been associated with suicidal behaviour. Moreover, health risk behaviours were found to be associated with suicidal behaviour, including inadequate physical activity and high sedentary behaviour,^14,15,16^ substance use (alcohol, tobacco and drugs),^17,18,19^ passive smoking,^20,21^ poor dietary behaviour, such as inadequate fruit and vegetable intake,^22^ and overweight or obesity.^23,24^

Based on data from the ‘World Mental Health Survey’, the prevalence of 12-month suicide ideation, plans and attempts were 2.1%, 0.7% and 0.4%, respectively, in 10 low- and middle-income countries,^2^ including lifetime prevalence of suicide attempts of 0.7% in Nigeria^25^ and 2.9% in South Africa.^26^ The 12-month prevalence of suicidal behaviour in a community sample in Ethiopia was 7.0% ideation, 4.6% planning and 3.7% attempt.^3^ Treatment seeking after a suicide attempt (among those with attempt) was 26.0% among community residents in Ethiopia.^3^ Globally, among community adult samples ‘34–42% of those with suicidal thoughts (i.e. suicide ideation or plan) received health care compared with 49–55% of those who made a suicide attempt’.^27^

The age-standardised suicide rates for all ages (per 100 000) were 11.3 per 100 000 in both sexes (17.5 among male sexes and 6.2 among female sexes) in Zambia in 2016, and in comparison 12.8 in both sexes (21.7 in male sexes and 5.1 in female sexes) in South Africa and 11.4 per 100 000 in lower middle-income countries.^28^ There is a lack of community-based data on non-fatal suicidal behaviour and its correlates in Zambia, a lower middle-income country in Southern Africa. Zambia has a total population of 17.4 million, life expectancy at birth is 53.6 years, 44.6% live in urban areas, 86.7% can read and write English and the major ethnic groups are Bemba 21%, Tonga 13.6%, Chewa 7.4%, Lozi 5.7%, Nsenga 5.3%, Tumbuka 4.4% and Ngoni 4%.^29^ This study aimed to assess the prevalence and correlates of the prevalence and correlates of ever suicide attempt and past 12-month suicidal behaviour (ideation, plan and/or attempt) among adults (18–69 years) in Zambia.

## Methods

Cross-sectional data were analysed from the nationally representative ‘2017 Zambia STEPS Survey’.^30^ A ‘multistage cluster sampling technique was used to select a nationally representative sample of adults in Zambia aged 18 to 69 years’.^31^

In the first stage of sampling, Standard Enumeration Areas (SEAs) were selected from each province using probability proportional to size (PPS), in the second stage, 15 households in rural SEAs and 20 households in urban SEAs were selected systematically using appropriate sampling interval based on the number of households in that SEA. (p. 23)

And in the third stage, one member from the eligible household members (18–69 years, residing in household) was selected by simple random sampling.^31^ After written informed consent was obtained from participants, field investigators gathered information by using an e-STEPS Android-based data collection tool.^31^ Field investigators were trained nurses, technologists, students and Central Statistical Office staff.^31^ Main field work started on 22 July 2017 and ended on 15 October 2017.^31^ The ‘overall response rate was 74.3%’.^31^

### Measures

*Outcome variables.* Suicidal behaviour was assessed with the ‘World Health Organization (WHO) STEPwise approach to surveillance – Mental Health-Suicide module’,^32^ including suicidal ideation, suicide plan and suicide attempt in the past 12 months, ever suicide attempt, method of suicide and care seeking (details in Supplementary Table 1).^32^

**TABLE 1:** Sample and suicidal behaviour characteristics among 18–69-year-old persons in Zambia.

Variable	Sample		Ever suicide attempt		Suicidal behaviour (past 12 months)†	
	*N*	%	*N*	%	*N*	%
**Socio-demographics**						
**All**	4302	-	99	2.3	368	8.5
**Age (years)**	-	-	13	1.5	61	6.6
50–69	832	19.3	-	-	-	-
35–49	1288	29.9	21	1.4	103	7.4
25–34	1157	26.9	33	2.7	100	8.9
18–24	1025	23.8	32	3.2	104	10.0
**Gender**						
Male	1614	49.0	35	1.9	100	5.8
Female	2688	51.0	64	2.8	268	11.1
**Education**						
< Primary	1546	28.8	31	2.0	145	10.1
Primary	1036	23.0	20	2.3	80	7.3
> Primary	1717	48.2	48	2.5	143	8.2
**Marital status**						
Married or cohabiting	2627	58.9	51	1.9	206	7.5
Single or separated or divorced or widowed	1665	41.1	48	2.9	161	10.0
**Employment status**						
Employed	2147	50.3	41	1.9	159	7.1
Non-paid	852	21.0	20	2.1	96	12.5
Unemployed	1296	28.8	38	3.2	113	8.1
**Residence**						
Urban	1642	48.8	53	2.8	175	9.7
Rural	2660	51.2	46	1.9	193	7.4
**Ethnic group**						
Bemba	1260	32.8	34	2.8	133	9.9
Tonga	1230	33.7	27	1.8	94	7.8
Chewa or Lozi	301	6.8	6	2.2	30	9.0
Other	1177	26.8	28	2.2	80	6.8
**Psychosocial distress**						
Alcohol use-related family problem	529	14.7	18	2.8	75	14.1
Family member died from suicide	252	6.2	11	6.2	39	15.8
**Health status and risk behaviours**						
Systolic blood pressure (< 100 mmHg)	152	3.4	5	3.0	14	11.3
**Body mass index**						
Normal	2716	69.0	65	2.5	225	8.1
Underweight	261	6.8	3	0.9	26	9.1
Overweight	687	16.7	17	2.0	64	9.8
Obesity	333	7.6	7	2.9	29	8.4
Diabetes	266	6.2	4	0.8	20	5.7
Raised total cholesterol	344	7.4	5	1.6	32	10.2
Heart attack, angina or stroke	160	3.0	11	8.1	27	18.8
Daily smoking	356	9.0	19	4.7	38	9.8
Daily smokeless tobacco	119	2.1	5	2.7	26	19.7
Passive smoking	1034	26.3	30	2.8	128	12.6
Alcohol dependence	242	7.4	11	3.3	40	15.2
Inadequate fruit and vegetable intake	4057	9.04	84	2.4	318	8.7
**Physical activity**						
Low	869	19.5	21	3.4	72	9.5
Moderate	449	13.3	12	1.7	58	12.3
High	2517	67.2	59	2.3	199	7.8
Sedentary behaviour	408	8.9	10	1.8	41	9.8

† , Past 12-month suicidal ideation (7.8%) and/or suicide plan (3.6%) and/or suicide attempt (1.1%).

*Socio-demographic* information included work status, marital status, age, sex and education.

*Psychosocial distress* indicators consisted of passive smoking, family member died from suicide and alcohol use-related family problem (details in Supplementary Table 1).^31^

*Health status and health risk behaviour* variables included blood pressure measurement (average of the last two of three readings), body mass index (‘measured < 18.5 kg/m^2^ underweight, 18.5–24.4 kg/m^2^ normal weight, 25–29.9 kg/m^2^ overweight and ≥ 30 kg/m^2^ obesity’), measured diabetes (‘fasting plasma glucose levels ≥ 7.0 mmol/L; or using insulin or oral hypoglycaemic drugs’), elevated total cholesterol (TC) (‘fasting TC ≥ 5.0 mmol/L or currently on medication for raised cholesterol’); history of ‘heart attack or chest pain from heart disease (angina) or a stroke (cerebrovascular accident or incident)’, daily smoking, daily smokeless tobacco use, inadequate fruit and vegetable intake (< 5 servings per day), passive smoking at home, in closed spaces or at work in the past 30 days, and based on the ‘Global Physical Activity Questionnaire (GPAQ)’, ‘low, moderate or high physical activity’ and sedentary behaviour (≥ 8 h per day).^31^ Physical activity was categorised by the median metabolic equivalents (METs) of performed activities as low (‘total physical activity METs minutes per week is < 600’), moderate:

(‘3 or more days of vigorous-intensity activity of at least 20 minutes per day OR; 5 or more days of moderate-intensity activity or walking of at least 30 minutes per day OR; 5 or more days of any combination of walking, moderate or vigorous intensity activities achieving a minimum of at least 600 MET-minutes per week’). (p. 14)

and high:

(‘vigorous-intensity activity on at least 3 days achieving a minimum of at least 1500 METs minutes per week OR; 7 or more days of any combination of walking, moderate or vigorous intensity activities achieving a minimum of at least 3000 MET-minutes per week’.). (p. 14)

physical activity.^33,34^ *Alcohol dependence* was assessed by using three questions of the ‘Alcohol Use Disorder Identification Test=AUDIT’ (items 4–6), for example, ‘How often during the last year have you found that you were not able to stop drinking once you had started?’ Response options ranged from ‘0=never to 4=daily or almost daily’; total scores of 4 or more indicate alcohol dependence.^35^

### Data analysis

Statistical analyses were performed by using STATA software version 15.0 (Stata Corporation, College Station, Texas, USA). Data were weighted to make the sample representative of the target population (adults in Zambia aged 18–69 years).^31^ Unadjusted and adjusted logistic regressions were applied to estimate the predictors of two outcomes: (1) ever having attempted suicide and (2) suicidal behaviour in the form of suicidal ideation, suicide plan and/or suicide attempt in the past year. Variables significant in unadjusted analysis were included in the multivariable logistic regression model. Only complete cases were included in the analysis. A *p*-value of < 0.05 was considered significant.

### Ethical consideration

The study was approved by the University of Zambia (UNZA) Research Ethics Committee (REC), and written informed consent was obtained from participants. The study was approved by the University of Zambia (UNZA) Research Ethics Committee (REC), and written informed consent was obtained from the participants.

## Results

### Descriptive data on the sample and suicidal behaviour

The study participants consisted of 4302 adults (median = 31 years, interquartile range (IQR) 18, range 18–69 years), 49.0% were male, 50.3% were employed, almost half (48.2%) had more than primary education and 58.9% were married or cohabiting. More than one in four participants (26.3%) were exposed to passive smoking, 6.2% had a close family member who died from suicide and 14.7% had alcohol use-related family problems. Almost one in four respondents (24.3%) was overweight or obese, 3.4% had a systolic blood pressure of less than 100, 6.2% had diabetes, 7.4% raised TC, 3.0% ever had a heart attack, angina or stroke, 9.0% smoked daily, 2.1% had smokeless tobacco daily, 90.4% had inadequate fruit and vegetables, 7.4% were dependent on alcohol, 8.9% were sedentary and 19.5% had low physical activity. Almost a hundred participants (2.3%) had ever attempted suicide and 8.5% engaged in past 12-month suicidal behaviour (ideation 7.8%, plan 3.6% and/or attempt 1.1%) (see Table 1).

The main methods used in the last suicide attempt were ‘razor, knife or other sharp instrument’ (19.7%), ‘poisoning with pesticides (e.g. rat poison, insecticide, weed killer)’ (17.4%), ‘hang on a rope’ (17.3%) and ‘overdose of medication (e.g. prescribed, over-the counter)’ (16.6%). About one in four participants (23.9%) sought medical care for the last suicide attempt, of which 71.4% were admitted to hospital overnight. Of those participants who reported past 12-month suicidal ideation (7.8%), 23.8% had sought professional help.

### Associations with ever suicide attempt and past 12-month suicidal behaviour

In adjusted logistic regression analysis, having a family member who died from suicide, having had a heart attack, angina or stroke and daily tobacco smoking were associated with ever suicide attempt.

In adjusted logistic regression, female sex, non-paid work status (including student, homeworker and retired), alcohol use-related family problem, passive smoking, heart attack, angina or stroke and alcohol dependence were positively associated with past 12-month suicidal behaviour, and belonging to other ethnic groups was negatively associated with past 12-month suicidal behaviour. In addition, in an unadjusted logistic regression analysis, 18–24-year-old participants, those who were never married, separated, divorced or widowed, having urban residence, family members died from suicide, having lower systolic blood pressure and daily smokeless tobacco use, were associated with past 12-month suicidal behaviour (see Table 2).

**TABLE 2:** Associations with ever suicide attempt and past 12-month suicidal behaviour.

Variable	Ever suicide attempt				Suicidal behaviour (past 12 months)†			
	COR	95% CI	AOR	95% CI	COR	95% CI	AOR	95% CI
**Socio-demographics**								
**Age (years)**								
50–69	1	Reference	-	-	1	Reference	1	Reference
35–49	0.90	0.39, 2.08	-	-	1.12	0.79, 1.60	1.15	0.74, 1.79
25–34	1.81	0.84, 3.92	-	-	1.37	0.95, 1.99	1.25	0.83, 1.88
18–24	2.12	0.99, 4.56	-	-	1.56	1.06, 2.31*	1.17	0.75, 1.83
**Gender**								
Male	1	Reference	-	-	1	Reference	1	Reference
Female	1.49 ()	0.88, 2.52	-	-	2.02	1.52, 2.69***	2.33	1.69, 3.22***
**Education**								
< Primary	1	Reference	-	-	1	Reference	-	-
Primary	1.10	0.58, 2.11	-	-	0.70	0.48, 1.03	-	-
> Primary	1.23	0.70, 2.15	-	-	0.80	0.58, 1.09	-	-
**Marital status**								
Married or cohabiting	1	Reference	-	-	1	Reference	1	Reference
Single or separated or divorced or widowed	1.51	0.92, 2.50	-	-	1.38	1.05, 1.83*	1.29	0.95, 1.77
**Employment status**		-						
Employed	1	Reference	-		1	Reference	1	Reference
Non-paid	1.07	0.54, 2.13	-	-	1.86	1.34, 2.60***	1.74	1.15, 2.61**
Unemployed	1.68	0.94, 3.02	-	-	1.15	0.84, 1.58	1.08	0.74, 1.58
**Residence**								
Urban	1	Reference	-	-	1	Reference	1	Reference
Rural	0.68	0.41, 1.11	-	-	0.74	0.56, 0.98*	1.03	0.75, 1.41
**Ethnic group**								
Bemba	1	Reference	-	-	1	Reference	0.71	0.50, 1.03
Tonga	0.63	0.33, 1.19	-	-	0.77	0.55, 1.08	0.78	0.46, 1.32
Chewa or Lozi	0.77	0.30, 2.02	-	-	0.90	0.54, 1.52	0.64	0.43 0.96*
Other	0.78	0.48, 1.43	-	-	0.66	0.45, 0.98*	-	-
**Psychosocial distress**								
Alcohol use-related family problem	1.24	0.64, 2.40	-	-	2.01	1.44, 2.81***	1.61	1.04, 2.48*
Family member died from suicide	3.19	1.34, 7.57**	3.01	1.20, 7.57*	2.17	1.36, 3.47***	1.58	0.95, 2.60
**Health status and risk behaviours**								
Systolic blood pressure	0.99	0.97, 1.00	-	-	0.99	0.98, 1.00*	1.00	0.99, 1.01
**Body mass index**								
Normal	1	Reference	-	-	1	Reference	-	-
Underweight	0.34	0.10, 1.21	-	-	1.13	0.67, 1.92	-	-
Overweight	0.81	0.42, 1.56	-	-	1.23	0.83, 1.84	-	-
Obesity	1.17	0.44, 3.11	-	-	1.04	0.59, 1.82	-	-
Diabetes	0.34	0.10, 1.11	-	-	0.62	0.36, 1.08	-	-
Raised total cholesterol	0.62	0.18, 2.11	-	-	1.20	0.73, 1.98	-	-
Heart attack, angina or stroke	4.01	1.94, 8.29***	4.00	1.84, 8.70***	2.60	1.53, 4.40	2.83	1.55, 5.16***
Daily smoking	2.35	1.23, 4.48**	2.36	1.21, 4.62*	1.18	0.76, 1.84	-	-
Daily smokeless tobacco	1.19	0.43, 3.26	-	-	2.72	1.57, 4.71***	1.76	0.87, 3.18
Passive smoking	1.32	0.77, 2.25	-	-	1.91	1.43, 2.57***	1.86	1.34, 2.58***
Alcohol dependence	1.50	0.67, 3.36	-	-	2.07	1.36, 3.15***	2.21	1.24, 3.9**
Inadequate fruit and vegetable intake	1/07	0.49, 2.33	-	-	1.17	0.74, 1.86	-	-
**Physical activity**								
Low	1	Reference	-	-	1	Reference	-	-
Moderate	0.50	0.20, 1.20	-	-	1.34	0.83, 2.16	-	-
High	0.67	0.34, 1.30	-	-	0.81	0.57, 1.16	-	-
Sedentary behaviour	0.81	0.38, 1.73	-	-	1.15	0.75, 1.75	-	-

COR, crude odds ratio; AOR, adjusted odds ratio; CI, confidence interval.

*** , *p* < 0.001;

** , *p* < 0.01;

* , *p* < 0.05.

† , Past 12-month suicidal ideation (7.8%) and/or suicide plan (3.6%) and/or suicide attempt (1.1%).

## Discussion

The investigation aimed to estimate the prevalence and correlates of ever suicide attempt and past 12-month suicidal behaviour (ideation, plan and/or attempt) among adults in Zambia. The prevalence of lifetime suicide attempt (2.3%) was higher than in previous studies in Nigeria (0.7%)^25^ and Bhutan (0.7%),^13^ but similar to South Africa (2.9%),^26^ and the prevalence of past 12-month suicidal ideation (7.8%), plan (3.6%) and attempt (1.1%) was higher than in 10 low- and middle-income countries (ideation 2.1%, plan 0.7% and attempt 0.4%),^2^ but similar to a low-resourced community in Ethiopia (ideation 7.0%, plan 4.6% and attempt 3.7%).^2^ The high rate of suicidal behaviour in Zambia is also confirmed in a previous study among adolescents in Zambia, with a prevalence of past 12-month suicidal ideation of 31.1%.^36^ The suicide rate in Zambia (11.3 per 100 000) was similar to the rate in lower middle-income countries (11.4 per 100 000).^28^

In this study, we found that female sex, having a family member who died from suicide and having an alcohol use-related family problem increased the odds for suicidal behaviour and/or ever suicide attempt. The main methods used in the last suicide attempt were using a sharp instrument, pesticides, rope and medication overdose, which are similar to suicides in low-resourced countries.^1^ By knowing the relevant suicide methods used, relevant prevention strategies can be devised, such as restricting access to pesticides.^1^ In this study, of those participants who reported past 12-month suicidal ideation, 23.8% had sought professional help, which is lower than globally among community adult samples (34%–42%),^27^ and among participants with a suicide attempt, 23.9% sought medical care which is lower than globally among community adult samples (49%–55%).^27^ One way of improving the access rate of treatment for suicidal behaviour and/or depression is by improving or expanding primary mental healthcare.^37,38^ A study among primary healthcare workers in Zambia showed that their ability to detect mental health problem early was limited and that additional in-service training was needed to improve their service delivery.^39^ Apart from primary healthcare screening for depression and suicidal ideation, for people not consulting primary care, community-level case-finding strategies may increase detection and management.^3^

In consistence with previous research,^2,3,13^ this study found that female sex, those whose work status was non-paid and in unadjusted analysis, younger age (18–24 year olds), those who were single, separated, divorced or widowed and having urban residence were associated with suicidal behaviour. However, in a review of studies in Africa, some studies reported a male or female predominance of suicide attempts, but other studies did not find sex differences.^40^ We did not find significant differences in the prevalence of suicidal behaviour in terms of educational level, while some previous studies^2,3,4,13^ showed associations between lower socio-economic or educational status and suicidal behaviour. Compared with persons belonging to the major ethnic groups (Bemba, Tonga, Chewa or Lozi), persons belonging to smaller ethnic groups in Zambia were less likely to report past 12-month suicidal behaviour. Further research is needed to explore reasons for these differences in the prevalence of recent suicidal behaviour by ethnic groups.

In line with former research findings on the family history of suicide,^2,3,13^ this study found an association between family members died from suicide and ever suicide attempt and in unadjusted analysis with past 12-month suicidal behaviour. In this study, a high proportion of participants reported family members died from suicide (6.2%). For survivors of suicide loss, peer support may be beneficial rather than help from friends or family members.^41^

In agreement with previous investigations,^1,12,17,18,19,20,21^ this study showed alcohol use-related family problems, alcohol dependence, passive smoking and daily smoking increased the suicidal behaviour, and in unadjusted analysis, smokeless tobacco use increased the odds for suicidal behaviour. The WHO estimates that the risk of suicide attempt increases manifold after alcohol consumption.^42^ This study showed that a high proportion of the general population were alcohol-dependent (7.4%). Alcohol misuse may increase both depression and adverse life events and may thus increase suicide attempts.^43^ Bang et al.^20^ have described several possible mechanisms by which passive smoking is associated with suicidal behaviour. For example, passive and firsthand smoking have similar physiological effects in increasing depression and suicide risk by decreasing hormones and neurotransmitters.^20^ However, as this study is cross-sectional and also did not assess the duration of passive smoking exposure, we cannot draw causative conclusions on the relationship between passive smoking and suicidal behaviour.

Having had a heart attack, angina or stroke (cardiovascular disease) increased the odds for suicidal behaviour. Similar results were found in the previous research.^6,44^ It is possible that an increase in disability in patients with cardiovascular diseases leads to an increase in suicidal behaviour.^37,44^ This study found in an unadjusted analysis that lower blood pressure (by using a continuous measure) was associated with past 12-month suicidal behaviour. In a large study in the general adult population in Korea, low systolic blood pressure (< 100 millimetre of mercury [mmHg]) showed a significant association with suicidal ideation.^10^ When using the same cut-off for low systolic blood pressure (< 100 mmHg) in this study, the prevalence of ever suicide attempt and past 12-month suicidal behaviour was higher, but not significantly higher. Further research, probably with larger sample sizes should explore the possible relationship between low blood pressure and suicidal behaviour. Unlike some previous research findings,^7,8,9,14,15,16,22,23,24^ this investigation did not show a significant association between diabetes, raised TC, overweight or obesity, inadequate physical activity, sedentary behaviour, insufficient fruit and vegetable intake and suicidal behaviour.

Compared with some studies on suicidal behaviour in other countries, a high prevalence of suicidal behaviour was found in Zambia. The findings of this study can inform mental healthcare providers who screen for suicide risk. For example, the higher rate of suicidal behaviour among women, persons with a non-paid work status, those who have psychosocial distress, who are dependent on alcohol and who have cardiovascular disorders should be addressed by healthcare providers when screening and treating Zambians who might be suicidal.

## Study limitations

Because of the cross-sectional study approach, it is not possible to establish a causative relationship between study variables and suicidal behaviour. Some of the variables were assessed by self-report, which may have resulted in biased responses. Furthermore, the 2017 Zambia STEPS survey did not assess other mental disorders, such as depression, and parental psychopathology. Some variables, such as household income, were not included in the analysis because of too many missing values.

## Conclusion

In this nationally representative sample of adults in Zambia, almost 1 in 10 had engaged in suicidal behaviour in the past 12 months, and 2.3% had ever attempted suicide. Several risk factors for suicidal behaviour and/or ever suicide attempt were identified, including female sex, non-paid work status, alcohol use-related family problems, passive smoking, family members died from suicide, daily tobacco smoking, heart attack, angina or stroke and alcohol dependence, which can assist in guiding interventions to prevent suicidal behaviour in the Zambian population.

## References

[1] World Health Organization (WHO) Suicide [homepage on the Internet]. 2019 [cited 2020 Aug 5]. Available from: https://www.who.int/news-room/fact-sheets/detail/suicide

[2] Borges G, Nock MK, Haro Abad JM, et al Twelve-month prevalence of and risk factors for suicide attempts in the World Health Organization World Mental Health Surveys. J Clin Psychiatry. 2010;71(12):1617–1628. 10.4088/JCP.08m04967blu 20816034 PMC3000886

[3] Jordans M, Rathod S, Fekadu A, et al Suicidal ideation and behaviour among community and health care seeking populations in five low- and middle-income countries: A cross-sectional study. Epidemiol Psychiatr Sci. 2018;27(4):393–402. 10.1017/S2045796017000038 28202089 PMC5559346

[4] Scottish Government Social Research Risk and protective factors for suicide and suicidal behaviour: A literature review [homepage on the Internet]. 2008 [cited 2020 Aug 5]. Available from: https://dspace.stir.ac.uk/bitstream/1893/2206/1/Suicide%20review%5b1%5d.pdf

[5] Scott KM, Hwang I, Chiu WT, et al Chronic physical conditions and their association with first onset of suicidal behavior in the world mental health surveys. Psychosom Med. 2010;72(7):712–719. 10.1097/PSY.0b013e3181e3333d 20498290 PMC12980531

[6] Moazzami K, Dolmatova EV, Feurdean M Suicidal ideation among adults with cardiovascular disease: The national health and nutrition examination survey. Gen Hosp Psychiatry. 2018;51:5–9. 10.1016/j.genhosppsych.2017.12.001 29268167

[7] Cheah YK, Azahadi M, Phang SN, Abd Manaf NH Sociodemographic, lifestyle and health determinants of suicidal behaviour in Malaysia. Psychiatry Res. 2018;261:319–324. 10.1016/j.psychres.2017.12.086 29331849

[8] Elamoshy R, Bird Y, Thorpe LU, Moraros J Examining the association between diabetes, depressive symptoms, and suicidal ideation among Aboriginal Canadian peoples living off-reserve: A cross-sectional, population-based study. Diabetes Metab Syndr Obes. 2018;11:767–780. 10.2147/DMSO.S184058 30538514 PMC6254537

[9] Daray FM, Mann JJ, Sublette ME How lipids may affect risk for suicidal behavior. J Psychiatr Res. 2018;104:16–23. 10.1016/j.jpsychires.2018.06.007 29920417 PMC6102068

[10] Joung KI, Cho SI Association of low blood pressure with suicidal ideation: A cross-sectional study of 10,708 adults with normal or low blood pressure in Korea. BMC Public Health. 2018;18(1):200 10.1186/s12889-018-5106-5 29490622 PMC5831223

[11] Angelakis I, Gillespie EL, Panagioti M Childhood maltreatment and adult suicidality: A comprehensive systematic review with meta-analysis. Psychol Med. 2019;49(7):1057–1078. 10.1017/S0033291718003823 30608046 PMC6498789

[12] Sorsdahl K, Stein DJ, Williams DR, Nock MK Associations between traumatic events and suicidal behavior in South Africa. J Nerv Ment Dis. 2011;199(12):928–933. 10.1097/NMD.0b013e3182392c39 22134450 PMC4148626

[13] Dendup T, Zhao Y, Dorji T, Phuntsho S Risk factors associated with suicidal ideation and suicide attempts in Bhutan: An analysis of the 2014 Bhutan STEPS survey data. PLoS One. 2020;15(1):e0225888 10.1371/journal.pone.0225888 31999708 PMC6991943

[14] Uddin R, Burton NW, Maple M, Khan SR, Tremblay MS, Khan A Low physical activity and high sedentary behaviour are associated with adolescents’ suicidal vulnerability: Evidence from 52 low- and middle-income countries. Acta Paediatr. 2020;109(6):1252–1259. 10.1111/apa.15079 31709627

[15] Vancampfort D, Stubbs B, Mugisha J, et al Leisure-time sedentary behavior and suicide attempt among 126,392 adolescents in 43 countries. J Affect Disord. 2019;250:346–353. 10.1016/j.jad.2019.03.053 30877857

[16] An KO, Jang JY, Kim J Sedentary behavior and sleep duration are associated with both stress symptoms and suicidal thoughts in Korean Adults. Tohoku J Exp Med. 2015;237(4):279–286. 10.1620/tjem.237.279 26596898

[17] Breet E, Goldstone D, Bantjes J Substance use and suicidal ideation and behaviour in low- and middle-income countries: A systematic review. BMC Public Health. 2018;18(1):549 10.1186/s12889-018-5425-6 29699529 PMC5921303

[18] O’Neill S, O’Connor RC Suicide in Northern Ireland: Epidemiology, risk factors, and prevention. Lancet Psychiatry. 2020;7(6):538–546. 10.1016/S2215-0366(19)30525-5 32006466

[19] Goldstone D, Bantjes J, Nel D, Stanbridge J, Lewis I Alcohol use predicts emergency psychiatric unit admission for non-fatal suicidal behaviour in the Western Cape (South Africa): A case-control study. Int J Psychiatry Clin Pract. 2020;24(2):163–172. 10.1080/13651501.2019.1711419 31928103

[20] Bang I, Jeong YJ, Park YY, Moon NY, Lee J, Jeon TH Secondhand smoking is associated with poor mental health in Korean adolescents. Tohoku J Exp Med. 2017;242(4):317–326. 10.1620/tjem.242.317 28867706

[21] Weng SC, Huang JP, Huang YL, Lee TS, Chen YH Effects of tobacco exposure on perinatal suicidal ideation, depression, and anxiety. BMC Public Health. 2016;16:623 10.1186/s12889-016-3254-z 27448804 PMC4957348

[22] Nanri A, Mizoue T, Poudel-Tandukar K, et al Dietary patterns and suicide in Japanese adults: The Japan public health center-based prospective study. Br J Psychiatry. 2013;203(6):422–427. 10.1192/bjp.bp.112.114793 24115342

[23] Brewer-Smyth K, Cornelius M, Pohlig RT Childhood adversity and mental health correlates of obesity in a population at risk. J Correct Health Care. 2016;22(4):367–382. 10.1177/1078345816670161 27742859 PMC5504522

[24] Henriksen CA, Mather AA, Mackenzie CS, Bienvenu OJ, Sareen J Longitudinal associations of obesity with affective disorders and suicidality in the Baltimore epidemiologic catchment area follow-up study. J Nerv Ment Dis. 2014;202(5):379–385. 10.1097/NMD.0000000000000135 24727724

[25] Gureje O, Kola L, Uwakwe R, Udofia O, Wakil A, Afolabi E The profile and risks of suicidal behaviours in the Nigerian survey of mental health and well-being. Psychol Med. 2007;37(6):821–830. 10.1017/S0033291707000311 17349104

[26] Joe S, Stein DJ, Seedat S, Herman A, Williams DR Non-fatal suicidal behavior among South Africans : Results from the South Africa stress and health study. Soc Psychiatry Psychiatr Epidemiol. 2008;43(6):454–461. 10.1007/s00127-008-0348-7 18473134 PMC2754160

[27] Bruffaerts R, Demyttenaere K, Hwang I, et al Treatment of suicidal people around the world. Br J Psychiatry. 2011;199(1):64–70. 10.1192/bjp.bp.110.084129 21263012 PMC3167419

[28] World Health Organization. Suicide in the world Global health estimates [homepage on the Internet]. 2019 [cited 2020 Aug 5]. Available from: https://apps.who.int/iris/bitstream/handle/10665/326948/WHO-MSD-MER-19.3-eng.pdf

[29] World Factbook [homepage on the Internet] Zambia [cited 2020 Aug 5]. Available from: https://www.cia.gov/library/publications/the-world-factbook/geos/print_za.html

[30] World Health Organization (WHO) STEPwise approach to surveillance (STEPS) [homepage on the Internet]. 2018 [cited 2020 Aug 5]. Available from: https://www.who.int/ncds/surveillance/steps/en/

[31] Republic of Zambia, Ministry of Health Zambia STEPS for non-communicable disease risk factors [homepage on the Internet]. 2017 [cited 2020 Aug 5]. Available from: https://www.who.int/ncds/surveillance/steps/Zambia-NCD-STEPS-Survey-Report-2017.pdf?ua=1

[32] World Health Organization (WHO) STEPwise approach to surveillance- mental health_suicide module [homepage on the Internet]. 2020 [cited 2020 Aug 5]. Available from: https://www.who.int/ncds/surveillance/steps/riskfactor/modules/en/

[33] Armstrong T, Bull F Development of the World Health Organization Global Physical Activity Questionnaire (GPAQ). J Pub Health. 2006;14:66–70. 10.1007/s10389-006-0024-x

[34] World Health Organization (WHO) Global Physical Activity Questionnaire (GPAQ) analysis guide. Geneva: World Health Organization; 2012.

[35] Australian Government Alcohol screen (AUDIT) [homepage on the Internet]. [cited 2020 Aug 5]. Available from: http://nceta.flinders.edu.au/files/3314/2257/4957/Right_Mix_3.pdf

[36] Muula AS, Kazembe LN, Rudatsikira E, Siziya S Suicidal ideation and associated factors among in-school adolescents in Zambia. Tanzan Health Res Bull. 2007;9(3):202–206. 10.4314/thrb.v9i3.14331 18087900

[37] Bachmann S Epidemiology of suicide and the psychiatric perspective. Int J Environ Res Public Health. 2018;15(7):1425 10.3390/ijerph15071425 PMC6068947 29986446

[38] Munakampe MN Strengthening mental health systems in Zambia. Int J Ment Health Syst. 2020;14:28 10.1186/s13033-020-00360-z 32322298 PMC7161303

[39] Chilema AM, Mwape L Factors affecting the ability of health care providers to detect mental problems at primary health care level: A case of Lusaka Community District Health Centres (LCDHC). Int J Med Health Res [serial online]. 2017;3(11):45–53. Available from: www.medicalsciencejournal.com

[40] Mars B, Burrows S, Hjelmeland H, Gunnell D Suicidal behaviour across the African continent: A review of the literature. BMC Public Health. 2014;14:606 10.1186/1471-2458-14-606 24927746 PMC4067111

[41] Bartone PT, Bartone JV, Violanti JM, Gileno ZM Peer support services for bereaved survivors: A systematic review. Omega (Westport). 2019;80(1):137–166. 10.1177/0030222817728204 28871835

[42] World Health Organization (WHO) Global status report on alcohol and health [homepage on the Internet] 2018 [cited 2020 Aug 5]. Available from: https://apps.who.int/iris/bitstream/handle/10665/274603/9789241565639-eng.pdf?ua=1&ua=1

[43] Brady J The association between alcohol misuse and suicidal behaviour. Alcohol Alcohol. 2006;41(5):473–478. 10.1093/alcalc/agl060 16891335

[44] Eriksson M, Glader EL, Norrving B, Asplund K Poststroke suicide attempts and completed suicides: A socioeconomic and nationwide perspective. Neurology. 2015;84(17):1732–1738. 10.1212/WNL.0000000000001514 25832661 PMC4424126

